# Introduction and spread of *vancomycin-resistant Enterococcus faecium* (VREfm) at a German tertiary care medical center from 2004 until 2010: a retrospective whole-genome sequencing (WGS) study of the molecular epidemiology of VREfm

**DOI:** 10.1186/s13756-024-01379-4

**Published:** 2024-02-14

**Authors:** Aila Caplunik-Pratsch, Bärbel Kieninger, Veronika A. Donauer, Johanna M. Brauer, Vanessa M. K. Meier, Corinna Seisenberger, Anca Rath, Daniel Loibl, Anja Eichner, Jürgen Fritsch, Wulf Schneider-Brachert

**Affiliations:** Department of Infection Prevention and Infectious Diseases, University Hospital Regensburg, Franz-Josef-Strauß-Allee 11, 93053 Regensburg, Germany

**Keywords:** Vancomycin-resistant *Enterococcus faecium*, VRE, Epidemiology, Germany, MLST, cgMLST, Infection control, Outbreak

## Abstract

**Background:**

In most of Europe and especially in Germany, there is currently a concerning rise in the number of hospital-acquired infections due to vancomycin-resistant *Enterococcus faecium* (VREfm). Therefore, there is a need to improve our understanding of the way VREfm spreads in hospitals. In this study, we investigated the molecular epidemiology of VREfm isolates from the first appearance at our university hospital in 2004 until 2010. There is only very scarce information about the molecular epidemiology of VREfm from this early time in Germany.

**Methods:**

Our analysis includes all available first VREfm isolates of each patient at our tertiary care center collected during the years 2004–2010. If available, additional consecutive VREfm isolates from some patients were analyzed. We used multilocus sequence typing (MLST) and core genome multilocus sequence typing (cgMLST) for the analysis and description of nosocomial transmission pathways as well as the detection of outbreaks.

**Results:**

VREfm isolates from 158 patients and 76 additional subsequent patient isolates were included in the analysis. Until 2006, detections of VREfm remained singular cases, followed by a peak in the number of VREfm cases in 2007 and 2008 with a subsequent decline to baseline in 2010. MLST and cgMLST analysis show significant changes in the dominant sequence types (STs) and complex types (CTs) over the study period, with ST192 and ST17 being responsible for the peak in VREfm cases in 2007 and 2008. The four largest clusters detected during the study period are comprised of these two STs. Cluster analysis shows a focus on specific wards and departments for each cluster. In the early years of this study (2004–2006), all analyzed VREfm stemmed from clinical specimens, whereas since 2007, approximately half of the VREfm were detected by screening. Of the 234 VREfm isolates analyzed, 96% had a *vanB* and only 4% had a *vanA* resistance genotype.

**Conclusions:**

This retrospective study contributes significant knowledge about regional VREfm epidemiology from this early VREfm period in Germany. One remarkable finding is the striking dominance of *vanB*-positive VREfm isolates over the entire study period, which is in contrast with countrywide data. Analysis of cgMLST shows the transition from sporadic VRE cases at our institution to a sharp increase in VRE numbers triggered by oligoclonal spread and specific outbreak clusters with the dominance of ST192 and ST17.

**Supplementary Information:**

The online version contains supplementary material available at 10.1186/s13756-024-01379-4.

## Introduction

While in the United States, vancomycin-resistant enterococci (VRE) infections in hospitalized patients already posed a significant problem in the 1990s, in Germany and most other European countries, VRE became a significant cause for hospital infections only 15–20 years later [1]. Unfortunately, recent EARS-Net surveillance data show an ongoing significant rise in vancomycin resistance for invasive *Enterococcus faecium* (VREfm) infections in the European Union (EU)/European Economic Area (EEA) [2]. Especially concerning is the sharp rise in the resistance rates in Germany: whereas in 2015, 10.5% of invasive *E. faecium* infections were caused by VRE, this number rose to 26.3% in 2019 [3].

Therefore, the question of how VREfm spreads so efficiently in hospitals is extremely relevant. One explanation points to the characteristics of hospital-associated (HA) clones of *E. faecium*: ampicillin resistance in addition to the intrinsic antibiotic resistances of enterococci, the potential to survive for prolonged periods on dry inanimate surfaces, tolerance of low concentrations of chlorine and an enhanced ability for biofilm formation and colonization [4–6]. A prerequisite for these described characteristics that favor survival in harsh hospital environments is a plastic genome that allows for the easy integration of new adaptive traits. In recent years, genomic research has contributed significantly to the current understanding of the development of these HA *E. faecium* lineages: *E. faecium* strains can be divided into a hospital-infection- and animal-associated lineage (clade A) and a community-associated lineage (clade B) [7]. Clade A can be further subdivided into clades A1 and A2, with clade A1 comprising the HA *E. faecium* lineages. Strains belonging to clade A1 are characterized by the ability to easily acquire and lose mobile genetic elements (including the *vanA* and *vanB* resistance clusters) and by common recombination events of the core genome. These events lead to new emerging clones, which may eventually establish new clonal clusters with the potential to replace circulating dominant clones on a regional, national, or sometimes even international level [7]. This phenomenon is reflected in the changing epidemiology of dominant sequence types (STs) and complex types (CTs) found in surveillance data of VREfm screening or clinical isolates reported in numerous studies [8–11].

For Germany, there is still a gap of knowledge concerning the molecular epidemiology of VREfm, especially before the significant rise in VRE rates that started in the 2010s. Overall, the time from 2000 to 2010 was characterized in Germany by the occurrence of regional outbreaks, especially in the southwestern part of Germany, while the general VRE prevalence was low [12]. On the other hand, the Paul Ehrlich Society observed that the proportion of *E. faecium* isolates that accounted for the overall number of enterococcal infections rose from 9.3% in 1998 to 41.4% in 2010 [13]. This phenomenon illustrates the success of *E. faecium* strains with special adaptations that allowed their spread, especially in the hospital environment. To a large extent, these *E. faecium* strains were still susceptible to vancomycin. However, an increase in the rate of vancomycin resistance from 2.7% in 2001 to 12.6% in 2010 [13] could also be observed. The German National Reference Center for staphylococci and enterococci (NRC) analyzed some selected early VREfm outbreaks by multilocus sequence typing (MLST), but epidemiological data of VRE in Germany based on whole-genome sequencing (WGS) from that period are very rudimentary.

In this study, we had the opportunity to investigate the emergence and accumulated occurrence of cases of VRE at our institution during an early phase. For this purpose, we investigated the molecular epidemiology of VREfm from 2004 to 2010 by performing a retrospective analysis of all available first VREfm isolates from each patient using WGS and characterization by MLST and core genome multilocus sequence typing (cgMLST). We chose this specific period because it covers the first advent of VREfm at our institution in 2004 and the subsequent rise and peak in the number of cases in 2008 followed by a decrease in the number of detected cases in 2009–2010.

To the best of our knowledge, this is the first study from that time in Germany that comprises a comprehensive analysis of the introduction and spread of VREfm at a tertiary care hospital using cgMLST analysis. Because cgMLST offers a much higher resolution than conventional MLST, it allows a more precise understanding of the way VREfm was introduced and then disseminated in our hospital.

## Methods

### Study design

This study was designed as a monocentric, descriptive retrospective analysis of all available first patient VREfm isolates from 2004 to 2010 at University Hospital Regensburg (UKR).

At our institution, we have collected and frozen all first VRE isolates of each patient during the study period 2004–2010 (and beyond). In practice, in addition to the first VRE isolate of each patient, for many patients, subsequent VRE isolates were also added to the VRE strain collection.

UKR is a tertiary care university hospital with 839 beds located in Regensburg, a city in Bavaria with a population of approximately 150,000 (in 2020). The hospital serves as a referral center for approximately 2.2 million people in the region of northeastern Bavaria.

### Laboratory procedures and molecular characterization

For clinical specimens for species identification, VREfm isolates were grown on sheep blood agar plates (Oxoid/Thermo Fisher Diagnostics GmbH, Wesel, Germany) for 24 h. Antimicrobial susceptibility testing of VRE was performed by VITEK II (bioMérieux, Inc., Durham, NC), and if isolates were found intermediate or resistant to vancomycin, further analysis with an agar diffusion test, according to Clinical and Laboratory Standards Institute (CLSI) guidelines valid at that time, was performed. In addition, we performed polymerase chain reaction (PCR) for *vanA* and *vanB* identification on each isolate [14]. For screening specimens, VRE hydrolysis of esculin in a broth (BBL Enterococcosel Broth, BD, France) with the addition of 6 ml/L vancomycin (Dr. Ebert, Germany) was used, and if found positive, triggered subsequent testing for species identification by PCR and additional *vanA* and *vanB* PCR testing. In general, all first VRE-positive specimens per patient were collected and frozen at -70 °C (Cryobank, MAST, Germany).

For this study, we thawed all frozen VRE isolates from 2004 to 2010 for WGS. Only isolates from inpatients or outpatients at our institution were included in this study. We registered basic patient data (birth, sex), date of the first positive specimen, and department, ward, and ward type (intensive care unit vs. normal ward). The type of specimen was categorized as wound, skin swab (or not otherwise specified), puncture, blood culture, catheter tip, urine, drainage/secretions, biopsy, BAL/sputum or other respiratory secretions and rectal swab (according to the categories of the NRC for enterococci [15]). We categorized specimens into possible infection or colonization according to the clinical material sent.

We performed testing for species identification with matrix-assisted laser desorption ionization time-of-flight mass spectrometry (MALDI-TOF MS; Bruker Daltonics) and for antibiotic resistance by BD Phoenix (phenotypic testing). Species other than VREfm were not included in further analysis.

WGS was performed by extraction of DNA with a QIAmp DNA Mini Kit (Qiagen Diagnostic GmbH, Germany). We measured DNA concentration and quality by Qubit (dsDNA HS array kit, Thermo Fisher Scientific, Germany). Sequencing libraries were generated using the Nextera XT library Prep Kit (Illumina, USA), and sequencing was performed on either MiniSeq oder NextSeq Dx550 (Illumina, USA) with a 2 × 150 bp paired-end sequencing run using either a high output (MiniSeq) or a mid-output cassette (NextSeq Dx 50).

Sequencing reads of this study with a mean assembled coverage depth of 124x (range 30–183) and a mean percentage of good targets of 99.0 (range 95.0–99.9) were further analyzed by cgMLST using SeqSphere + version 9.0.1 (Ridom GmbH, Münster, Germany).

We defined affiliation to a cluster as genotypes with a maximum difference of three alleles [16, 17]. We used cgMLST comparison for 158 first patient isolates and for 33 additional subsequent patient isolates that differed in the MLST and/or cgMLSType from the first patient isolate to create a neighbor joining tree in the newick format in SeqSphere+. Based on these data, we visualized the genetic relationship between the isolates using itol (interactive tree of life) version 5 [18].

We used the SeqSphere tools NCBI AMR Finder Plus for detecting genes conferring antimicrobial resistance and *E. faecium* virulence factor database (VFDB) for searching for virulence determinants (http://www.mgc.ac.cn/VFs/).

## Results

### Bacterial isolates and study cohort

Overall, we analyzed 234 VREfm isolates in this study belonging to 158 different patients (Table 1).

**Table 1:** Overview of sequenced VRE isolates at Regensburg University Hospital 2004–2010

	Total number of first VRE isolates of each patient	Number of sequenced first VRE isolates per patient (percentage of coverage of total number of first VRE isolates per patient)	Sequenced Isolates overall (including multiple isolates per patient)	Sequenced first VRE isolates per patient and year	Sequenced isolates from clinical material (including multiple isolates per patient)	Sequenced first VRE isolates per patient and year from clinical material	Percentage sequenced first VRE isolates from clinical material/sequenced first VRE isolates per patient and year (%)
Overall	195	158 (81.0%)	234	170	121	103	61
2004	3	1 (33.0%)	1	1	1	1	100
2005	13	10 (76.9%)	10	10	10	10	100
2006	13	12 (92.3%)	13	12	13	12	100
2007	63	47 (74.6%)	61	48	32	28	58
2008	58	50 (86.2%)	86	57	38	30	53
2009	27	25 (92.6%)	43	28	22	18	64
2010	18	13 (72.2%)	20	14	5	4	29

This means that for a large portion of patients, we analyzed more than one VREfm isolate.

**Fig. 1:**
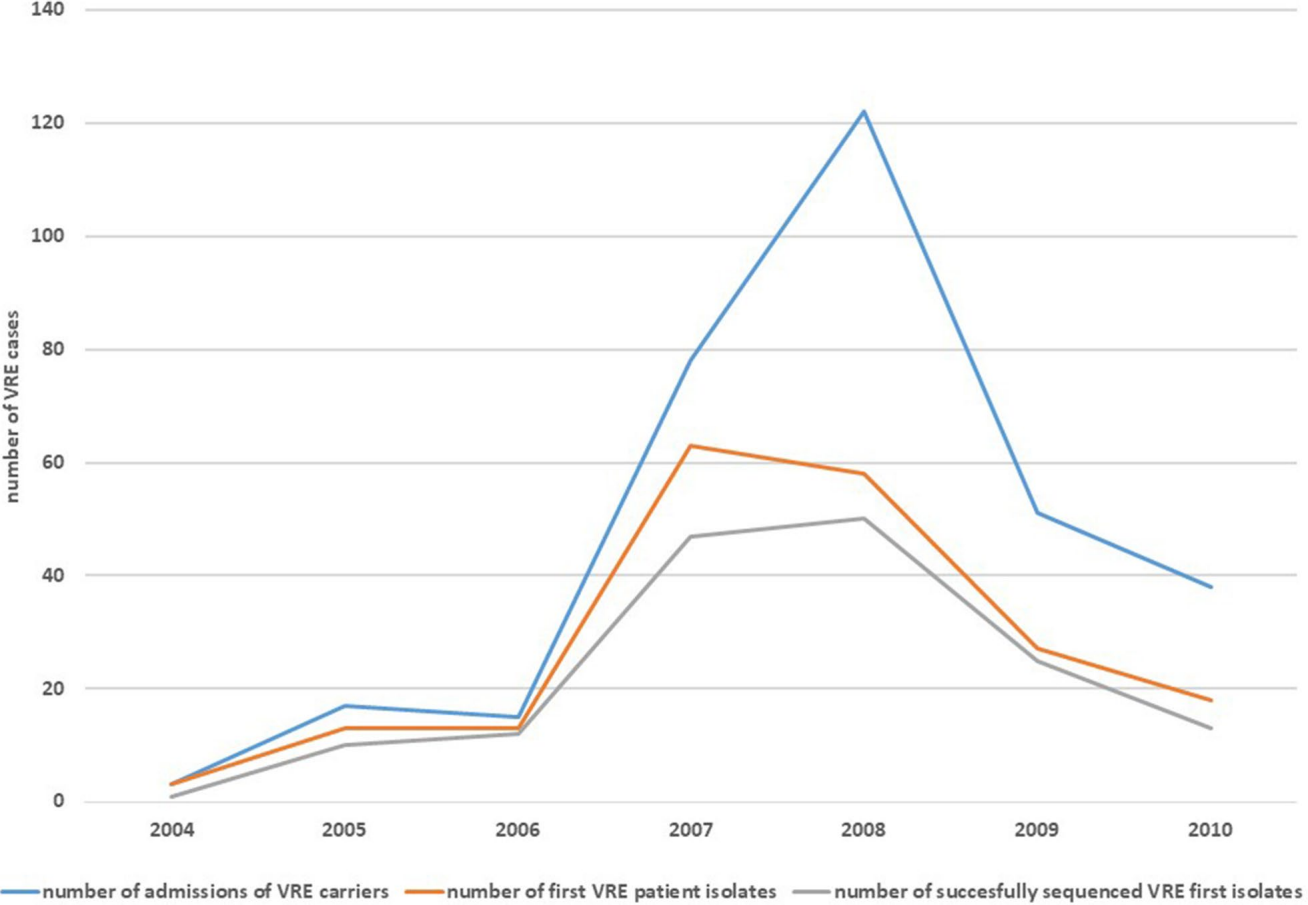
Development of VRE carrier admissions compared with VRE first patient isolates at Regensburg University Hospital 2004–2010. This figure shows the development of the number of hospital stays of VREfm carriers at our institution (blue line) during the study period, the number of first VREfm isolates of each patient according to surveillance data (orange line) and the number of successfully sequenced first VREfm isolates of each patient (gray line). The proportion of successfully sequenced first patient isolates of all first patient isolates is depicted as well and reaches 81% over the study period with little variation over time

According to existing surveillance data from our department, 195 patients with VREfm infection or carrier status were treated at our institution from 2004 to 2010, implying that our isolate collection represents approximately 81% (158/195) of VREfm patients from that time (for details, see Fig. 1 and Table 1). Furthermore, Table 1 shows that the rising number of VREfm cases at our institution reflected a rise in VREfm isolates stemming from clinical material as well. Nonetheless, the introduction of VREfm screening at our hospital in 2007 significantly influenced the percentage of the first VREfm isolates of each patient per year stemming from clinical material and not from screening. During the first three years of the study, none of the new VREfm isolates were screening isolates, whereas in 2010, the proportion of new VREfm isolates from clinical material decreased to only 29% (for details, see Table 1).

Figure 1 shows the development of VREfm cases at our institution: While the peak in new first VRE patient isolates was in 2007, the highest number of VRE carrier admissions was one year later. The number of hospital admissions of VREfm carriers is higher than the number of first VREfm isolates of each patient according to the surveillance data, meaning that patients on average have more than one hospital stay per year and that newly diagnosed VREfm carriers will often return for further hospital stays in the following year.

**Fig. 2:**
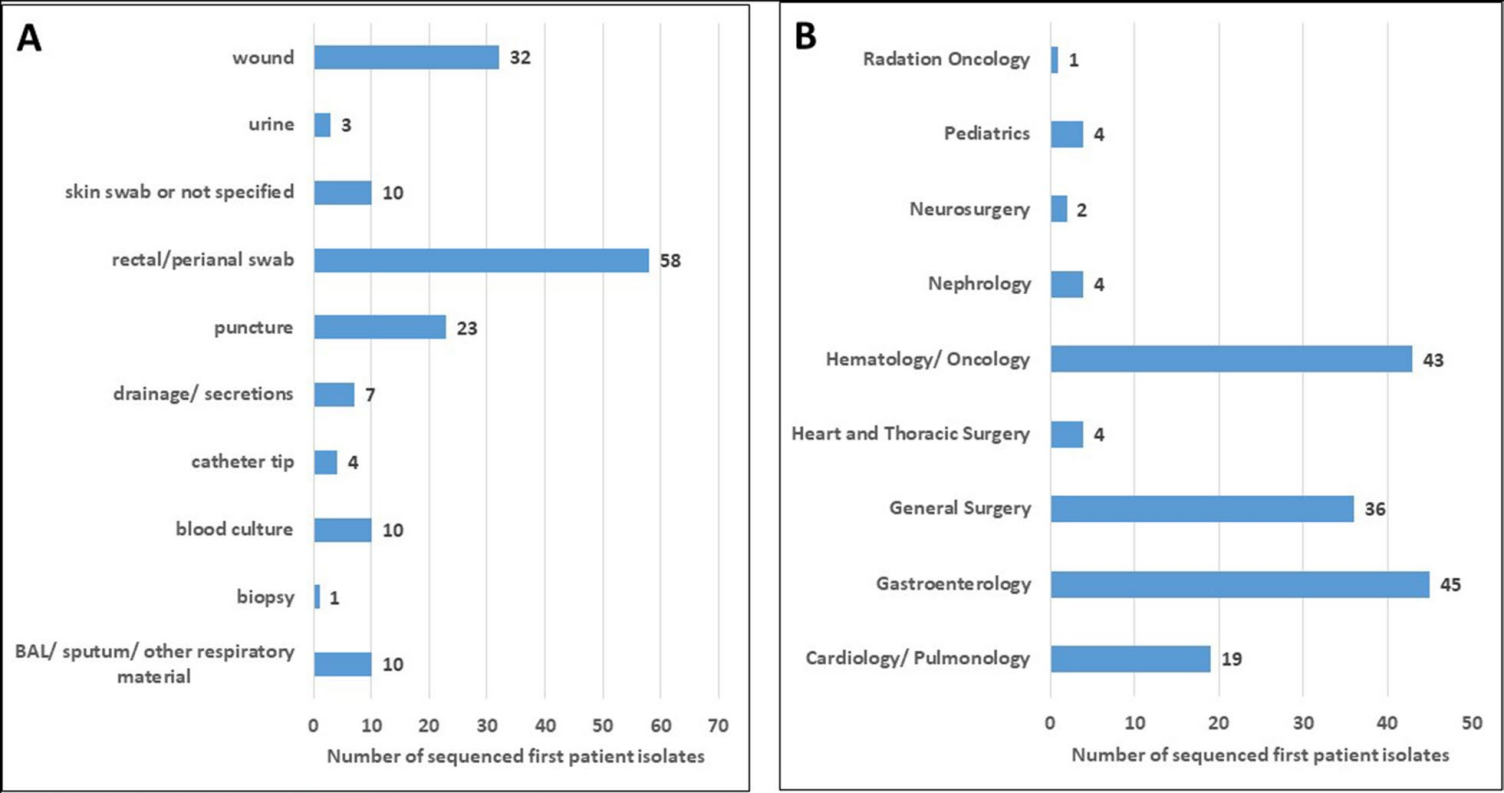
**A** Material sources of sequenced first patient isolates. **B** Distribution of the associated departments of the sequenced first patient isolates

The majority of the 158 available first VREfm isolates were recovered from rectal/perianal swabs (58; which means that 37% are screening isolates), followed by wound swabs (32) and puncture material (23) (shown in Fig. 2A). For the additional available 76 subsequent VREfm patient isolates, the vast majority were rectal/perianal screenings (54; 71% screening isolates). Most first patient isolates were detected in the gastroenterology department (28%), followed by the hematology/oncology department (27%) and the general surgery department (23%) (Fig. 2B).

### MLST and cgMLST analysis

**Fig. 3:**
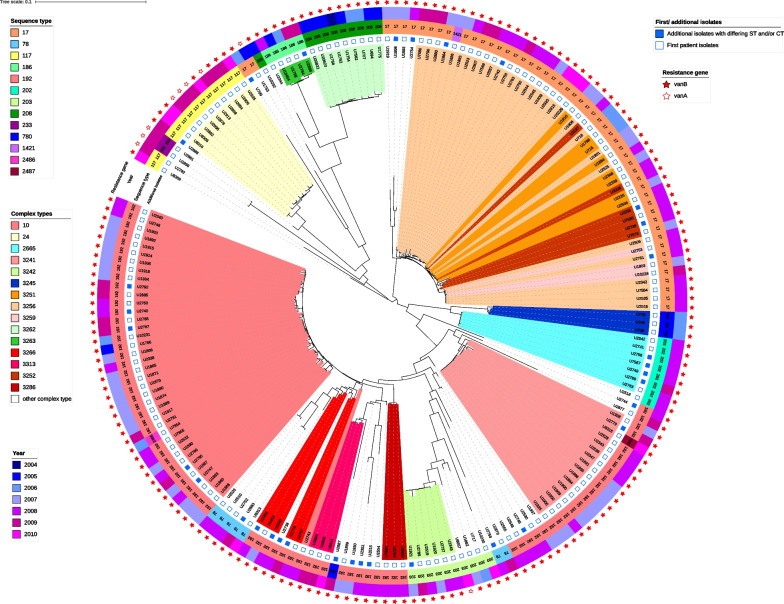
Genetic relationship of 191 study isolates (all first patient isolates and 33 additional subsequent isolates with differing MLST/cgMLSType) displayed as a circular midpoint rooted phylogenetic tree based on a Neighbor Joining Tree with distance based on MLST and cgMLST alleles. Read from inside to outside: The color of the inner circle and clade corresponds to a specific CT. CTs that were identified only once or twice are marked in white. In the first ring from inside isolates that are not first patient isolates but are included in this image as they differ in their MLST and/or cgMLSType are marked with a blue square. The next ring shows the 13 different sequence types which are marked with different colors. The outer ring of color strips shows the year of isolation, which visualizes the switch in the dominating STs and CTs over the years. The stars in the outer circle correspond to the resistance gene identified, thereby illustrating the dominance of *vanB* (red stars) in our study isolates and their preponderance for certain CTs

Overall, the isolates of our study (first and subsequent isolates) belong to 13 different STs, 11 previously known STs, and 2 newly defined STs (ST2486 and ST2487). Using the cgMLST scheme, the isolates could be further divided into 48 different CTs. The details of the genetic relationship of 191 isolates (all first patient isolates and 33 additional subsequent isolates with differing MLST/cgMLSType) are shown in Fig. 3. Furthermore, Fig. 3 shows the distribution of the CTs and STs of the isolates, their identified glycopeptide resistance genes, and the year of their isolation.

The frequency of each ST and CT differed in absolute numbers and changed significantly over the study time (Fig. 3 and Additional file 4: Fig. S1). In the first years of the study period from 2004 to 2006 and before the sharp rise in the number of VREfm cases in 2007, ST208 was the most common ST (10/23, 43%) and consisted mainly of CT3262 (9/10). The second and third were ST17 (4/23, 17%) and ST780 (3/23, 13%) (referring to the first VREfm isolates per patient and year).

In 2007 and 2008, ST192 was the most common ST (57/105, 54%), followed by ST17 (31/105, 30%). Together, these two STs comprised 84% of the first VREfm isolates during these two years.

**Fig. 4:**
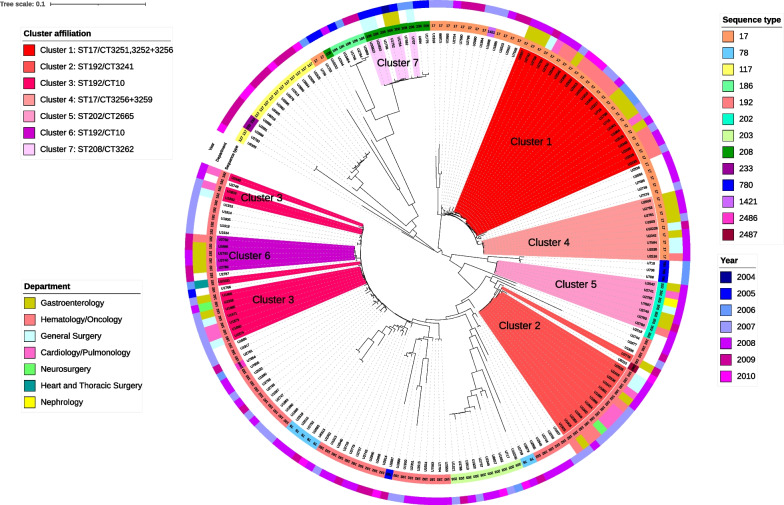
Cluster affiliation and department distribution of 191 study isolates (all first patient isolates and 33 additional subsequent isolates with differing MLST/cgMLSType) within the same phylogenetic tree presented in Fig. 3. Read from inside to outside: The colors of the clades correspond to the affiliated cluster of the isolates. The inner ring of color strips visualizes the STs of the isolates. The colors of the middle ring indicate the department of origin of the isolates belonging to the seven largest clusters. The color strips of the outer ring visualize the year of isolation

The peak in numbers of the most frequent sequence type ST192 in the years 2007–2008 is due to the spread of isolates belonging to the second (ST192/CT3241) and third (ST192/CT10) largest clusters detected during the study period (Fig. 4). The peak in numbers of the second most frequent ST ST17 in the years 2007–2008 can be assigned to the spread of the largest (ST17/with the 3 closely related complex types CT3251,3252 + 2356) and the fourth largest cluster (ST17/CT3256 + 3259) during the study period (Fig. 4).

In the years 2009–2010, the absolute numbers of VRE declined sharply at our institution. Parallel to this, the composition of the STs is also in transition: The most common ST (referred to first isolates per year) is ST117 (12/42, 29%), followed by ST17 (10/42, 24%) and ST192 (9/42, 21%). ST117 consists mostly of CT24 (10/12), and ST17 consists mainly of CT2356 (9/10), while ST192 is more heterogeneous (CT10:4/9, CT3313:3/9) (Additional file 4: Fig. S1).

### Cluster analysis

For cluster affiliation, we created a minimum spanning tree of 191 isolates by cgMLST comparison (including all first patient isolates of the study (158 isolates) and subsequent patient isolates that differed in their ST and/or CT from the first patient isolate (additional 33 isolates)). When applying a cutoff of ≤ 3 alleles difference for affiliation to a cluster and a minimum of three isolates for defining a cluster, we identified 11 distinct clusters during the study period. The genetic relationship of the isolates belonging to the seven largest clusters of the study as well as the year and department of their isolation are shown in Fig. 4.

In the detailed analysis, we found that isolates of 24 different patients belonged to cluster 1, the largest cluster identified in our study. All isolates of cluster 1 belonged to three closely related CTs (CT3251, CT3252 and CT3256) within sequence type 17. Isolates belonging to cluster 1 were collected from 2006 to 2010, with a peak in 2007 (7 cases) and 2008 (10 cases).

Among the isolates of cluster 1, we identified an accumulation of cases in the hematology/oncology department (12 patients) and in the gastroenterology department (five cases in the normal wards of that department, four cases in their associated medical intensive care unit (ICU)).

The second largest cluster comprises isolates stemming from 14 different patients, all isolates belonging to ST192/CT3241. The isolates were detected from 2007–2009, with a sharp peak in 2007 (8 isolates). We found an accumulation of cases in one specific medical ICU (4 cases) and in the hematology/oncology department (4 cases).

The third cluster comprises isolates from 11 different patients; all isolates belong to ST192/CT10. The time span in which isolates of this cluster were found is limited to 2006–2008, with a peak in 2007 (2007: 7 isolates). Four of the patients with VREfm belonging to cluster 3 were in the cardiology/pulmonology department at the time of detection, and three were in one specific surgical ICU.

The fourth cluster comprises isolates from nine different patients belonging to ST17/CT3256 and CT3259, all but two isolates from 2008. Three isolates were obtained from patients in the general surgery department, and three were obtained from patients in one medical ICU.

The fifth cluster comprises seven isolates of ST202/CT2665 collected between May 2008 and March 2009 in different departments of our hospital. The sixth and seventh clusters comprise 5 isolates each. Isolates of the sixth cluster belong to ST192/CT10 and were collected from 2008–2009 mainly in the gastroenterology department (4/5 isolates). The seventh cluster represents an early cluster of isolates belonging to ST208/CT3262 that were collected from September 2004 to July 2006 in the departments of general surgery and gastroenterology.

The other four clusters detected in the study comprise less than five patients each.

The notable concentration of cases in specific departments or wards of our hospital, as described above, implies that nosocomial transmissions lead to several small and medium-sized outbreaks, thereby triggering the spread of VRE. Due to incomplete patient screenings, however, the outbreaks that occurred can only be partially reconstructed.

### Antibiotic resistance mechanisms

#### Glycopeptide resistance

In the vast majority of isolates, *vanB* was detected (225/234; 96%). In only nine isolates overall from nine individual patients, *vanA* was detected (4%). Seven of these nine isolates were detected in 2009, 4 of them in ST117/CT24 (Fig. 3).

### Other antibiotic resistances and virulence factors

We found significant differences in the distribution of several antibiotic resistance genes between different STs (for more details see Additional file 1: Table S1). For example, *aph(2’’)-Ia* conferring high-level resistance to gentamicin was found only in 7% of all isolates, mainly in ST117/CT24. On the other hand, the *ant(6’)-Ia* gene conferring high-level resistance to streptomycin was often identified (72%); however, isolates belonging to ST192/CT10 typically lacked it. Details about the detected virulence factors are shown in Additional file 2: Table S2.

### Patients with multiple VRE isolates

Although the primary goal of our study was to analyze the first isolate of each patient, we also included 45 patients with multiple VREfm isolates (2.7 isolates on average). Interestingly, 25 of these 45 patients (55%) had differing STs and/or CTs in subsequent isolates. In Fig. 3, these differing subsequent isolates are marked with a blue square. The details of these isolates and the allele differences between the isolates are shown in Additional file 3: Table S3.

For some patients (marked in gray color in Additional file 3: Table S3), we found that they must be colonized (or infected) by different VRE strains simultaneously:

For patient 3, simultaneous colonization is likely because the first rectal isolate was characterized as ST17/CT3256, followed approximately three weeks later by the identification of a rectal isolate as ST192/CT10, while approximately one week later, two samplings (rectal and wound) taken on the same day identified each of these two different strains.

For patient 12, we found that on the same day, a rectal swab detected a VRE belonging to ST17/CT3252, while a vaginal swab detected a VRE of ST192/CT10.

For patient 23, two rectal screenings were performed on the same day detecting two different CTs (ST17/CT5126 and CT3259).

For patient 25, we detected over the course of about one month three times ST192/CT10 in a wound, while one time in the middle of this period at the same sampling site VRE ST192/CT3266 was detected.

We did not identify two different VRE strains from the same sampling site for any patient. The explanation for this could be, however, that it was practice for this study to pick only one colony of each preserved specimen for sequencing.

Apart from the four patients mentioned above, simultaneous colonization with more than one strain was not obvious, but because only one VREfm isolate was used for subculture, we cannot exclude that further patients in our study could have harbored more than one distinct ST/CT simultaneously.

## Discussion

During the study period from 2004 to 2010, a significant increase in detected VREfm isolates occurred at our tertiary care center in South Germany, Bavaria, peaking in 2007 and 2008. Despite a decline in VRE cases in the final two years of the study, a more pronounced surge in VREfm cases emerged from 2011 onwards, resulting in the current hyperendemic state.

Utilizing WGS, we retrospectively analyzed the molecular epidemiology of VREfm spread in our institution. Over the study period, we found a profound shift in detected STs and CTs: ST208 predominated initially (2004–2006), followed by ST17 and ST780. Likewise, Borgmann et al. identified ST208 alongside ST203 as one of the most common STs in a hospital in Baden Wuerttemberg from 2004 to 2005 [19]. Klare el al. [20], however, identified different prevalent STs in a collection of isolates from South West German hospitals from 2003 and 2004: 39% of VREfm isolates belonged to ST203 and 17% to ST192. Werner et al. [21] described that for 51 VREfm isolates that were send to the NRC from 2004 to 2006 from 19 hospitals in 10 federal states, the most common ST was ST18, followed by ST203.

In 2007–2008, the years of the first peak in the VRE numbers at our institution, ST192 and ST17 (58 and 31 first isolates per year) became the prevailing STs. Interestingly, ST192 spread in our hospital in several separate clusters comprised of CT3241 (cluster 2) and CT10 (clusters 3 and 6). By MLST analysis only, this pattern could not be recognized. Isolates of ST17, on the other hand, form the largest VREfm cluster observed at our hospital, which consists of several closely related CTs. However, within ST17, we could also identify another distinct cluster (cluster 4).

As described above, Klare et al. [20] found ST192 to be the second most common ST in VREfm isolates from South West German hospitals; unfortunately, there is no information about the CT. Furthermore, from 2011 to 2013, ST192 represented the second most prevalent ST identified as responsible for invasive VREfm infections by the NRC [12].

In our study, all ST192 isolates carried *vanB* as a resistance gene, which is in accordance with the findings of the NRC [12, 22]. For the first 30 non-outbreak VRE isolates at the Charité in 2008, WGS was performed and identified ST17 to be the most common ST, followed by ST192 [8]. In contrast to our ST192 isolates, all the ST192 isolates from that study belonged to CT164. In addition, ST17 was identified as one of the most common STs in isolates from one university hospital in East Bavaria from 2000 to 2004 [23]. Unlike that study where all the isolates were *vanA* positive, all but one of our ST17 isolates were *vanB* positive.

At the end of our study period (2009–2010), the new dominant ST was ST117, while the major outbreak strains of the former period, ST17 and ST192, decreased in number. We first detected ST117 at our hospital in 2009 and found that it is mainly comprised of CT24. The NRC found that ST117 was the most common ST detected in a collection of 69 chosen blood culture isolates from 2011 to 2012, followed by ST192 [15]. Interestingly, Werner et al. describe a spread of *vanA* ST117/CT24 as responsible for a countrywide spread of VRE in the 1990s and the disappearance of that CT at the end of that decade [12]. Nonetheless, CT24 is the predominant CT of our ST117 isolates that all originated from 2009 to 2010. Remarkably, we found both *vanA-* and *vanB-*positive CT24 isolates. A recent study from the Charité analyzing 120 VREfm isolates collected in 2008, 2013, 2015 and 2018 found that the percentage of VREfm of ST117 increased from 17% in 2008 to 57% in 2018 [8]. In 2018, ST117/CT71 comprised 13/30 VREfm isolates (43%), meaning that the majority of ST117 isolates belonged to only one specific CT. In the same study, 3% and 17% of the analyzed VREfm isolates from 2008 and 2013 were categorized as ST117/CT24, respectively [8].

In the analysis of the resistance genes and virulence determinants of the study isolates, we could identify typical patterns for different STs and CTs. However, we found no single or combination of differences in these genes that imply a clear advantage in the hospital environment and would thereby explain the change in the detected STs and CTs over the study period.

Another interesting finding of our study is that the portion of *vanA*-positive VREfm is very low throughout the study period. This is surprising considering that from 2004 to 2008, a clear majority of VREfm sent to the German NRC carried *vanA* [24]*.* During the same time, we detected only one single isolate at our institution that was *vanA* positive. In 2009 and 2010, the NRC identified for the first time similar numbers of *vanA-* and *vanB*-positive VREfm or even a slight overweight of *vanB* [24]. Data from Limbach Laboratory—a laboratory serving multiple hospitals in southwestern Germany—show that the resistance rate of *E. faecium* to vancomycin rose from 14% to approximately 31% from 2004 to 2010, while the resistance rate to teicoplanin remained stable at approximately 10% in those years, a pattern which implies a rise in the portion of *vanB*-positive VREfm [24]. Furthermore, the data show that from 2005 on, the rates of *vanB-* and *vanA*-positive VREfm at the Limbach laboratory were already similar, and from 2009 onwards, *vanB* was the predominant resistance gene. The explanation why *vanB* was the prevalent resistance cluster in VREfm at Regensburg University Hospital before it spread nationally is certainly connected to the distribution of certain STs and CTs in Regensburg, given that specific STs and CTs have a clear preponderance for either *vanA* or *vanB.*

Although it was not the primary focus of this study, for a significant portion of the patients, we included more than one VREfm isolate in the WGS analysis. The remarkable finding is that for more than half of the patients with two or more VREfm isolates, the identified STs and/or CTs were not identical. Our analysis of multiple patient isolates includes clinical isolates from different body sites as well as screening isolates. Therefore, the differences in the STs and CTs could imply a change in the colonizing VRE strain over time, for example, the substitution of one VRE strain by another strain with a fitness advantage or concomitant colonization or infection at the same body site by more than one VREfm strain. In cases of differing sampling sites, variations in the dominant VREfm strain depending on the body site are also possible. Several studies have shown concomitant carriage of more than one VREfm/VSEfm lineage by patients [25, 26]. Transposon analysis could determine whether and to what extent differing VREfm strains in the same patient were the result of transfer of the *van* gene on former vancomycin-sensitive *E. faecium* [27]*.* Recognizing slight differences in the appearance of *E. faecium* colonies suspicious of differing clones and therefore deciding to sequence more than one colony is challenging and rarely practiced. One study from Denmark [28] investigated whether for patients with invasive VREfm infections, the cgMLSType and/or the plasmid of the isolate causing the infection was identical to a preceding screening isolate within 60 days before infection. Of 19 VREfm pairs, 13 (68%) had a matching CT and plasmid, and 1 (5%) had a non-matching CT but a matching plasmid. The mismatches had a longer interval between colonization and infection (median 18 days) compared to the pairs with a cgMLST match (median 6 days). This suggests that generally, the colonizing strain is responsible for the invasive infection but that this relation can only be detected if the rectal screening is performed close to the infection, as the colonizing strains might change over time. Due to our seven-year study period, we had a very long observation time for patients with long or recurrent hospital stays, which might explain in part the observed high rate of changes in MLST and cgMLST.

Through cluster analysis, we could investigate the way VREfm was introduced and spread in our hospital. There is no real standard for defining the affiliation of isolates to a cluster. Abdelbary et al. investigated the relatedness of VREfm isolates of 156 patients by WGS over a period of three years and found that most isolates involved in outbreaks (91%) differed by 0 to 3 SNPs [16]. In a study about patient and environment interplay in the transmission of VRE, Correa-Martinez et al. first suggested using a cluster threshold for VREfm based on cgMLST differences of ≤ 3 alleles [17]. In line with this, we decided to use a cutoff of a maximum of three allele differences in the cgMLST comparison for affiliation with a cluster.

Our cluster analysis shows the spread in mostly small-medium size clusters that mainly have a focus on several wards or departments. For example, cluster 1 involves mainly the hematology/oncology and gastroenterology departments, including its associated ICU, while the focus of cluster 3 is the cardiology/pulmonology department and one specific surgical ICU.

Since 2007, it has become practice at our institution to screen contact patients of VRE carriers, and the hematology/oncology department introduced screening before hematopoietic stem-cell transplantation (HSCT). Apart from that, in the case of outbreaks or accumulation of VRE cases, we started to apply intensified screening strategies in certain wards. This implementation of screening is a significant influencing factor in our analysis. At that time, two consensus papers discussing the need for screening strategies were published, which represent the first German recommendations for VRE infection control [29, 30]. As Mutters and Frank have shown [31], the application of VRE screening may lead to a false impression of an increasing VRE burden even in times of decreasing VRE rates. In our case, a noteworthy number of VRE carriers were undoubtedly identified solely through the implementation of screening. On the other hand, the sharp rise in VRE-positive isolates stemming from clinical material in 2007 and 2008 proves that the spread of VRE in these years at our hospital is real and not a screening artifact.

In conclusion, investigating the molecular epidemiology of VREfm from the moment of its appearance at our hospital has highlighted several important findings, as there are very scarce data available about the molecular epidemiology of VREfm from that early time in Germany. In the few published studies, typing was usually performed using the inherent low discriminatory power of MLST only or other methods that do not allow for interinstitutional comparison, such as macrorestriction pattern analyses of genomic DNA resolved in pulsed field gel electrophoresis (PFGE).

We followed the path of the first VREfm introduced in our institution and observed significant changes in the dominant STs and CTs over the whole study period. On the other hand, our study identified that even in the beginning of the German VRE area, the prevalence and distribution of STs and CTs as well as *van*A and *van*B resistance clusters essentially reflect mainly regional or local circumstances. The distribution of the clusters identified by cgMLST are suggestive of small outbreaks in specific wards or departments, probably often reflecting introductions of different VREfm strains into our hospital with subsequent nosocomial dissemination. As screening was not performed at our institution during the first three years of the study and afterwards only in specific situations -mainly in the case of VRE accumulations-, the dissemination patterns of VREfm can obviously only be partly reconstructed. In addition, we often found differing STs and/or CTs for individual patients, which raises the question of whether sequencing only one isolate for one patient at a time is sufficient for infection control purposes.

As a significant outcome of our study results and to address the limitations posed by our screening policy's incomplete picture of VRE spread, we began sequencing all first VREfm isolates in 2015, initiating proactive genomic surveillance. This approach enables us to select and tailor infection prevention interventions more accurately.

### Supplementary Information

**Additional file 1**. **Table S1:** Additional resistance genes and their distribution within the 234 study isolates.

**Additional file 2**. **Table S2:** Virulence factors and their distribution within the 234 study isolates.

**Additional file 3**. **Table S3:** Overview and isolate details of patients with a change in the ST and/or CT of their sequenced VRE isolate over time.

**Additional file 4**. **Fig S1:** Change in the distribution of detected STs of first patient isolates over the study period.

## Data Availability

The datasets generated and analyzed during the current study are available from the corresponding author upon reasonable request.

## Abbreviations

cgMLST: Core genome multilocus sequence typing
CLSI: Clinical and Laboratory Standards Institute
CT: Complex type
EARS-Net: European Antimicrobial Resistance Surveillance Network
*E. faecium*: *Enterococcus faecium*
HA: Hospital-associated
HSCT: Hematopoietic stem-cell transplantation
ICU: Intensive care unit
MALDI-ToF MS: Matrix assisted laser desorption ionization time of flight mass spectrometry
MLST: Multilocus sequence typing
NRC: National reference center
PCR: Polymerase chain reaction
PFGE: Pulsed field gel electrophoresis
ST: Sequence type
UKR: University Hospital Regensburg
VRE: Vancomycin-resistant enterococci
VREfm: Vancomycin-resistant *Enterococcus faecium*
WGS: Whole-genome sequencing
