# Supplementary material for: Introduction and spread of vancomycin-resistant Enterococcus faecium (VREfm) at a German tertiary care medical center from 2004 until 2010: a retrospective whole-genome sequencing (WGS) study of the molecular epidemiology of VREfm
Source: Antimicrob Resist Infect Control. 2024 Feb 14;13:20. doi: 10.1186/s13756-024-01379-4 (PMC10865517; doi:10.1186/s13756-024-01379-4)
Supplement: Supplementary file 1 — Additional file 1. Table S1: Additional resistance genes and their distribution within the 234 study isolates. [file 13756_2024_1379_MOESM1_ESM.docx]

**Supplementary Table 1: Additional resistance genes and their distribution within the 234 study isolates**

| **Resistance gene** | **Distribution in percentage**  **of all 234 isolates** | **Findings regarding the**  **distribution in specific STs/CTs** |
| --- | --- | --- |
| *aph(2’’)-Ia* | 7% | mainly ST117/CT24 |
| *ant(6’)-Ia* | 72% | typically lacking in ST192/CT10 |
| *msrC* | 100% | species-specific gene for *E. faecium* |
| *ermB* | 90% |  |
| *ermA* | 4% |  |
| *dfr* | 39% | common in ST17 |
| *Inu(B)* | 28% | common in ST202 |
| *Isa(E)* | 36% | common in ST192 |
| *catTC* | 0.4% | only one isolate: ST117/CT5128 |
| *catA* | 3% | ST117/CT24 only |
| *tet(L)* | 24% | predominantly in ST192, ST780  and ST202 |
| *tet(M)* | 6% | predominantly in ST203 |
| *tet(W)* | 8% | predominantly in ST208 and ST186 |
