# Supplementary material for: Introduction and spread of vancomycin-resistant Enterococcus faecium (VREfm) at a German tertiary care medical center from 2004 until 2010: a retrospective whole-genome sequencing (WGS) study of the molecular epidemiology of VREfm
Source: Antimicrob Resist Infect Control. 2024 Feb 14;13:20. doi: 10.1186/s13756-024-01379-4 (PMC10865517; doi:10.1186/s13756-024-01379-4)
Supplement: Supplementary file 2 — Additional file 2. Table S2: Virulence factors and their distribution within the 234 study isolates. [file 13756_2024_1379_MOESM2_ESM.docx]

**Supplementary Table 2: Virulence factors and their distribution within the 234 study isolates**

| **Resistance gene** | **Distribution in percentage**  **of all 234 isolates** | **Findings regarding the distribution in specific STs/CTs** |
| --- | --- | --- |
| *acm* | 98% |  |
| *sgrA* | 97% |  |
| *ecbA* | 54% | lacking in almost all ST192 isolates except for one |
| *scm* | 0% |  |
