# Supplementary material for: Introduction and spread of vancomycin-resistant Enterococcus faecium (VREfm) at a German tertiary care medical center from 2004 until 2010: a retrospective whole-genome sequencing (WGS) study of the molecular epidemiology of VREfm
Source: Antimicrob Resist Infect Control. 2024 Feb 14;13:20. doi: 10.1186/s13756-024-01379-4 (PMC10865517; doi:10.1186/s13756-024-01379-4)
Supplement: Supplementary file 3 — Additional file 3. Table S3: Overview and isolate details of patients with a change in the ST and/or CT of their sequenced VRE isolate over time. [file 13756_2024_1379_MOESM3_ESM.docx]

**Supplementary Table 3: Overview of patients with a change in the ST and/or CT of their sequenced VRE isolates over time**

| **Patient number** | **Sequence type** | **Complex Type** | **Collection Date** | **Localization** | **vanA/**  **vanB** | **number of allele differences in cgMLST comparison** | |
| --- | --- | --- | --- | --- | --- | --- | --- |
| 1 | 780 | 3245 | 01.06.2006 | abscess anus | vanB | 336 |  |
| 1 | 17 | 3278 | 22.07.2006 | blood culture central line | vanB |  |  |
| 2 | 203 | 3242 | 22.07.2008 | rectal/perianal screening | vanB | 230 |  |
| 2 | 17 | 3256 | 14.08.2008 | blood culture | vanB |  |  |
| 3 | 17 | 3256 | 03.02.2009 | rectal/perianal screening | vanB | 173 |  |
| 3 | 192 | 10 | 27.02.2009 | rectal/perianal screening | vanB |  | 174 |
| 3 | 17 | 3256 | 05.03.2009 | rectal/perianal screening | vanB | 175 |  |
| 3 | 192 | 10 | 05.03.2009 | wound | vanB |  |  |
| 4 | 17 | 3256 | 02.06.2008 | rectal/perianal screening | vanB | 165 |  |
| 4 | 192 | 3274 | 20.08.2008 | rectal/perianal screening | vanB |  |  |
| 5 | 192 | 3241 | 01.02.2008 | tip of central line | vanB | 68 |  |
| 5 | 192 | 10 | 28.08.2008 | rectal/perianal screening | vanB |  |  |
| 6 | 203 | 3255 | 11.02.2007 | fluid from abdominal drain | vanB | 203 |  |
| 6 | 17 | 3256 | 02.03.2007 | ascites | vanB |  |  |
| 7 | 192 | 10 | 03.10.2007 | rectal/perianal screening | vanB | 165 |  |
| 7 | 78 | 5127 | 15.10.2008 | rectal/perianal screening | vanB |  |  |
| 8 | 17 | 3251 | 17.05.2008 | rectal/perianal screening | vanB | 30 |  |
| 8 | 17 | 3252 | 14.07.2008 | rectal/perianal screening | vanB |  | 146 |
| 8 | 202 | 2665 | 02.09.2008 | rectal/perianal screening | vanB |  |  |
| 9 | 203 | 3242 | 08.10.2007 | rectal/perianal screening | vanB | 6 |  |
| 9 | 203 | 3242 | 09.06.2008 | rectal/perianal screening | vanB |  | 203 |
| 9 | 202 | 2665 | 02.03.2009 | rectal/perianal screening | vanB | 190 |  |
| 9 | 78 | 3275 | 21.07.2009 | rectal/perianal screening | vanB |  |  |
| 10 | 117 | 5128 | 02.02.2009 | rectal/perianal screening | vanA | 257 |  |
| 10 | 192 | 10 | 27.03.2009 | rectal/perianal screening | vanB |  |  |
| 11 | 203 | 3242 | 16.02.2009 | rectal/perianal screening | vanB | 419 |  |
| 11 | 186 | 3263 | 27.08.2009 | rectal/perianal screening | vanB |  | 3 |
| 11 | 186 | 3263 | 03.11.2010 | rectal/perianal screening | vanB |  |  |
| 12 | 17 | 3256 | 21.02.2008 | vaginal | vanB | 16 |  |
| 12 | 17 | 3252 | 11.08.2008 | rectal/perianal screening | vanB |  | 175 |
| 12 | 192 | 10 | 11.08.2008 | vaginal | vanB |  |  |
| 13 | 17 | 3257 | 29.08.2007 | rectal/perianal screening | vanB | 48 |  |
| 13 | 17 | 3258 | 10.09.2007 | rectal/perianal screening | vanB |  |  |
| 14 | 192 | 3266 | 15.10.2008 | bile | vanB | 174 |  |
| 14 | 202 | 2665 | 20.11.2008 | rectal/perianal screening | vanB |  |  |
| 15 | 192 | 3313 | 06.08.2009 | urine | vanB | 79 |  |
| 15 | 780 | 3312 | 20.08.2009 | rectal/perianal screening | vanB |  | 182 |
| 15 | 117 | 3288 | 27.08.2009 | rectal/perianal screening | vanB | 1 |  |
| 15 | 117 | 3288 | 15.10.2009 | rectal/perianal screening | vanB |  |  |
| 16 | 192 | 3241 | 16.09.2007 | rectal/perianal screening | vanB | 75 |  |
| 16 | 192 | 3266 | 16.01.2008 | rectal/perianal screening | vanB |  |  |
| 17 | 192 | 10 | 09.11.2008 | wound | vanB | 178 |  |
| 17 | 17 | 3256 | 18.11.2008 | wound | vanB |  |  |
| 18 | 192 | 3241 | 28.09.2007 | rectal/perianal screening | vanB | 159 |  |
| 18 | 192 | 3306 | 23.11.2007 | rectal/perianal screening | vanB |  | 65 |
| 18 | 192 | 3307 | 07.01.2008 | rectal/perianal screening | vanB | 78 |  |
| 18 | 192 | 3308 | 13.03.2008 | rectal/perianal screening | vanB |  |  |
| 19 | 192 | 3274 | 16.06.2008 | rectal/perianal screening | vanB | 0 |  |
| 19 | 192 | 3274 | 18.07.2008 | rectal/perianal screening | vanB |  | 71 |
| 19 | 192 | 26 | 21.08.2008 | rectal/perianal screening | vanB |  |  |
| 20 | 192 | 10 | 23.11.2007 | wound | vanB | 1 |  |
| 20 | 192 | 10 | 23.11.2007 | tracheal secretions | vanB |  | 153 |
| 20 | 202 | 2665 | 08.10.2008 | wound | vanB |  |  |
| 21 | 17 | 3259 | 17.09.2007 | rectal/perianal screening | vanB | 231 |  |
| 21 | 203 | 3242 | 08.10.2007 | rectal/perianal screening | vanB |  | 0 |
| 21 | 203 | 3242 | 12.10.2007 | rectal/perianal screening | vanB |  |  |
| 22 | 17 | 3256 | 12.03.2008 | sputum | vanB | 155 |  |
| 22 | 192 | 3266 | 27.05.2008 | rectal/perianal screening | vanB |  | 16 |
| 22 | 192 | 5125 | 28.07.2008 | rectal/perianal screening | vanB |  |  |
| 23 | 17 | 5126 | 22.09.2008 | rectal/perianal screening | vanB | 24 |  |
| 23 | 17 | 3259 | 22.09.2008 | rectal/perianal screening | vanB |  |  |
| 24 | 192 | 3310 | 10.07.2008 | tip of central line | vanB | 112 |  |
| 24 | 192 | 3241 | 12.01.2009 | genital | vanB |  | 73 |
| 24 | 192 | 10 | 20.04.2009 | rectal/perianal screening | vanB |  |  |
| 25 | 192 | 10 | 17.12.2008 | wound | vanB | 16 |  |
| 25 | 192 | 10 | 03.01.2009 | wound | vanB |  | 37 |
| 25 | 192 | 3266 | 04.01.2009 | wound | vanB | 37 |  |
| 25 | 192 | 10 | 23.01.2009 | wound | vanB |  |  |
