# Supplementary material for: Introduction and spread of vancomycin-resistant Enterococcus faecium (VREfm) at a German tertiary care medical center from 2004 until 2010: a retrospective whole-genome sequencing (WGS) study of the molecular epidemiology of VREfm
Source: Antimicrob Resist Infect Control. 2024 Feb 14;13:20. doi: 10.1186/s13756-024-01379-4 (PMC10865517; doi:10.1186/s13756-024-01379-4)
Supplement: Supplementary file 4 — Additional file 4. Fig S1: Change in the distribution of detected STs of first patient isolates over the study period. [file 13756_2024_1379_MOESM4_ESM.pptx]

## Slide 1
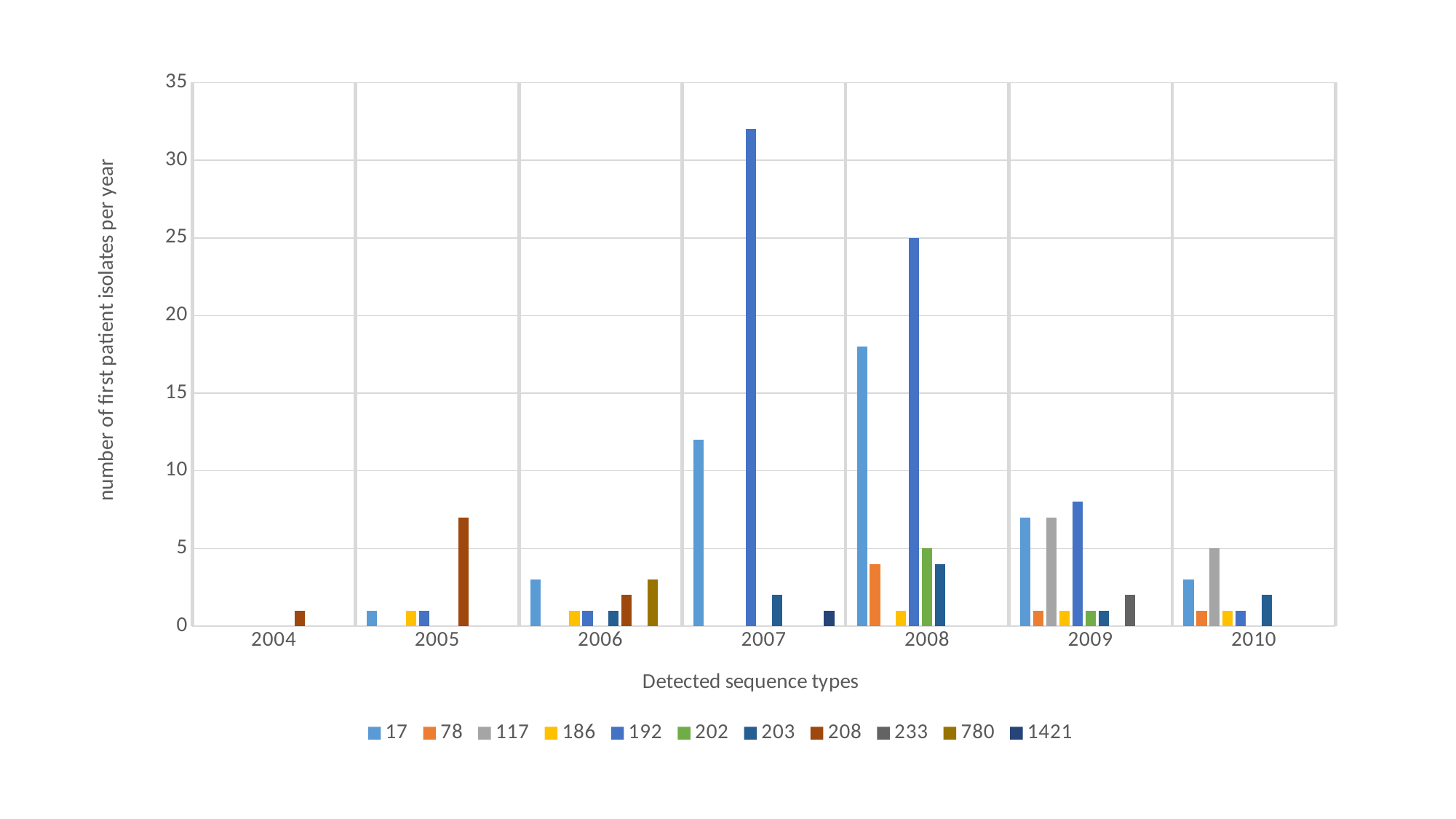

### Chart
| Category | 17 | 78 | 117 | 186 | 192 | 202 | 203 | 208 | 233 | 780 | 1421 |
|---|---|---|---|---|---|---|---|---|---|---|---|
| 2004 | None | None | None | None | None | None | None | 1.0 | None | None | None |
| 2005 | 1.0 | None | None | 1.0 | 1.0 | None | None | 7.0 | None | None | None |
| 2006 | 3.0 | None | None | 1.0 | 1.0 | None | 1.0 | 2.0 | None | 3.0 | None |
| 2007 | 12.0 | None | None | None | 32.0 | None | 2.0 | None | None | None | 1.0 |
| 2008 | 18.0 | 4.0 | None | 1.0 | 25.0 | 5.0 | 4.0 | None | None | None | None |
| 2009 | 7.0 | 1.0 | 7.0 | 1.0 | 8.0 | 1.0 | 1.0 | None | 2.0 | None | None |
| 2010 | 3.0 | 1.0 | 5.0 | 1.0 | 1.0 | None | 2.0 | None | None | None | None |
